# The Status of the Inlay in Comparison with Other Fillings

**Published:** 1900-11-15

**Authors:** Joseph Head

**Affiliations:** Philadelphia, Pa.


### THE STATUS OF THE INLAY IN COMPARISON WITH OTHER FILLINGS.

BY JOSEPH HEAD, D D.S , PHILADELPHIA, PA.

Read before the Dental Society of the State of New York, May 9, 1900, and reprinted from the *Dental Cosmos.*

Before considering the status of inlays let us first examine the characteristics of the perfect filling, then by a comparative table of all the filling materials the good and bad points of each may be justly ascertained. The characteristics of the ideal filling may be named as follows: (1) Strength sufficient to withstand mastication, (2) resistance to action of oral fluids, (3) color to match the tooth, (4) exclusion of bacteria and preclusion from growth of those that enter the margins, (5) non-conductivity of heat, (6) manipulation easy to patient, (7) manipulation easy to dentist, (8) manipulation inexpensive to healthy tooth structure.

Cohesive gold possesses the sole and important superiority over all other materials of greatest edge strength and resistance to the crushing force of mastication. True, it excludes bacteria from the cavity, but experience proves that if ever the edge of a cohesive gold filling begins to leak micro-organisms, the cohesive gold seems to be almost entirely lacking in the antiseptic power possessed by tin, gutta-percha and the cements. And, while it resists perfectly the action of the oral fluids, it is so utterly lacking in the other ideal attributes of good color, non-conductivity of heat, ease of patient, ease of dentist and manipulation inexpensive to tooth structure, that in the case of front teeth, soft teeth, or teeth of nervous patients its manifest objections more than counterbalance its advantages.

Soft gold is open to the same objections, while it has the advantages of resistance to wear, resistance to oral fluids, and, to a less degree, exclusion of bacteria. It also seems to possess within itself a power of inhibiting the growth of germs to such an extent that it is often advantageously combined with cohesive gold to make fillings that possess the advantages of both.

Tin has to a marked degree the good and bad attributes of soft gold, but it turns black.

Amalgam bulges under mastication, chips on the edges, rusts and leaks, but through antiseptic action it precludes decay. It is a moderately poor conductor of heat when rusty, and is easy of adaptation for the patient, easy for the dentist and calls for a manner of manipulation that is conservative of healthy tooth structure. Therefore, it is often available where gold is not.

Oxyphosphate of zinc has all the advantages wanting in cohesive gold with the exception of color, and lacks all the good points that cohesive gold possesses. Its edge strength is solely due to its great adhesion. It wears under mastication, dissolves in the fluids of the mouth and usually absorbs bacteria; but, on the other hand, it prevents the growth of germs, is a non-conductor of heat, has better color than gold, is easy of insertion for patient, easy of insertion for dentist, preserves weak walls and does not require undercuts.

The same may be said of oxychlorid of zinc, except that it causes pain to sensitive dentin and exposed gums.

Gutta-percha in a similar way, with the exception of color, possesses the good points lacking in cohesive gold, and lacks the good points possessed by cohesive gold. It loses shape and wears under mastication, has poor resistance to fluids of the mouth, poor color and leaks micro-organisms; but, on the other hand, it inhibits the germs that enter from further growth, it is a non-conductor of heat, is easy of insertion for both patient and dentist and has a manipulation that tends to conserve frail though healthy walls.

Now, when we come to inlays we have a filling in which the good points of the cement are combined with those of amalgam, gold or porcelain in such a way as to insure the advantages of both in the largest degree and reduce to a minimum the disadvantages of each. For when a cavity is lined with a thin zinc cement which is squeezed out by the insertion of soft amalgam, this amalgam afterward being dried of its mercury, and the edges of the metal burnished to the cavity margins, there is to all intents and purposes an inlay of amalgam. This treatment takes away from the amalgam three of its serious objections—conductivity of heat, lack of adhesion to the cavity and discoloration of adjacent tooth structure—while the adaptation of the plastic metal to the tooth margins can be quite as perfect as though no cement were used. So in using the principle of the inlay with amalgam three distinct advantages are gained without any counteracting disadvantages, all of which would seem to indicate that whenever possible zinc cement should be used under amalgam.

When an inlay of gold is cemented into a cavity by oxyphosphate of zinc all the advantages of cohesive gold and oxyphosphate of zinc are obtained, except that a fine line of cement remaining at the margins may in time prove a source of weakness. Such a filling, compared with the ideal filling, possesses excellent resistance to mastication, resistance—except in the fine line above mentioned—to the action of oral fluids, excludes bacteria or precludes the growth of those that enter, is a non-conductor of heat and entails a manipulation easy for the patient, while the manipulation of the practitioner does not involve greater hardship or loss of tooth structure than is called for in the use of cohesive gold. Therefore, the two objections that can be raised against this filling are bad color and an edge protected by a soluble cement. Hence we must conclude, given a firm tooth structure that can bear the mallet without danger, a dentin not sensitive to thermal changes, and a patient not too severely prostrated by the necessary malleting, a cohesive gold filling is superior to a gold inlay, inasmuch as the cohesive filling may have edges that perfectly exclude bacteria, even though it has no antiseptic action on germs The inlay, though largely inhibiting from growth the germs that enter its margin, does nevertheless allow them to enter, and the filling that keeps out the germs entirely must be held superior to the one that lets in the germs and then destroys them. Unfortunately, in the soft, sensitive teeth of nervous patients the manipulation of cohesive gold does not result in the exclusion of decay germs. The tooth margins may be powdered or weakened in some way by the manipulation or apposition of the gold, and the entering germs cause rapid decay, the cohesive gold not having the antiseptic power of restraining them. The thermal shocks and the overwrought condition of the patient, that sometimes last longer than we could wish, all tend to produce unhealthy conditions of the mouth, and consequent tooth dissolution. In such a mouth the inlay is preferable. The patient should not be made to undergo the malleting. As the germs of decay will probably enter under any circumstances, it is necessary to use a filling whose action will inhibit or prevent their growth.

In approximal cavities so situated that the filling does not show, or where great resistance to the blows of mastication is necessary, the gold inlay is usually to be preferred. Its sole objection is the fine line of cement that connects it with the cavity walls; but if the gold inlay is properly prepared, burnished and finished, the line of cement may be rendered so microscopic as to become practically no longer a source of danger. In other respects the gold inlay, when not visible, approaches very nearly the requirements of the ideal filling, having perfect resistance to mastication, power to preclude the growth of bacteria, non-conductivity of heat, ease of manipulation, firm adherence to cavity walls and an adaptation not usually so destructive to tooth structure as is the ordinary insertion of a gold filling.

The porcelain inlay in labial and buccal cavities possesses more nearly than any other class the characteristics of the ideal filling. Such inlays may have color that really matches the tooth. They may be set with a cellulose cement that is nearly, if not quite, insoluble in the oral fluids; that excludes germs of decay and precludes the growth of those that enter. A porcelain inlay is a non conductor of heat, adheres to cavity walls, has manipulation easy to patient and inexpensive to tooth structure. The only real drawback to labial porcelain fillings lies in the great skill and patience required in their insertion. Where, however, porcelain inlays have to withstand a severe strain of mastication, they demand the use of oxyphosphate of zinc as a cement, and are apt to chip on the edges. This objection, therefore, makes them somewhat less serviceable for non-visible approximal cavities in molars and bicuspids than gold inlays. For while the porcelain is sufficiently strong to withstand the crushing force of mastication, the chipping cf the margins tends to accentuate the weakness already found in the solubility of the cement that is their sole defense against bacteria. If such chipping occurs on the masticating surface of the molars or bicuspids, the fractured margins may readily be filled with gold in such a way that edge strength equal to gold is obtained. Also if the edges of the inlays are painted with insoluble cellulose before the filling is cemented into place, insolubility is added to the strength and adhesibility of the oxyphosphate of zinc. So with care and patience the guarded porcelain inlay acquires the advantages of gold, cement and porcelain, with none of the usual defects. For the porcelain fillings properly guarded may possess the natural color that no other filling material possesses, strength to withstand mastication, resistance to the fluids of the mouth, power to exclude bacteria and to inhibit the growth of those that enter, non-conductivity of heat and adaptability not excessively expensive of tooth structure. And if at times the manipulation for the dentist is of necessity so deft and artistic that the highest skill and judgment are required, we should be glad for our own sakes that such development and effort are demanded, that dentistry is so steadily advancing to that position where practice and theory are united in perfection.

### DISCUSSION OF DR. CAPON'S AND DR. HEAD'S PAPER.

DR. JOHN I. HART: An all porcelain crown is preferable to one that is only faced with porcelain. The later, to be at all resistant to the force of mastication, must be tipped with metal, this treatment in all instances rendering the crown less satisfactory esthetically, and not any stronger than an all-porcelain crown.

Unquestionably, the weakness of a porcelain inlay lies in the cement that is used to retain it, and when a portion of the filling is sheltered this danger is magnified. Eliminate this weakness; in other words, produce a cement that is insoluble in the oral fluids, and the dental millennium will have been reached.

DR. WILLIAM D. TRACY: With a certain class of practitioners esthetic considerations seem to have but little weight, and with another class the tendency seems to be to sacrifice the practical to the esthetic. But if we so cultivate our discrimination that we may strike the happy medium, we will find in porcelain a most valuable addition to the methods now in use.

From my observation it seems that the antiseptic action of amalgam, generally speaking, is not evident, and that recurrent decay takes place around a defective amalgam filling as quickly and as surely as it does at the margin of any other filling of similar environment.

Gutta-percha is mentioned as having poor resistance to the fluids of the mouth. In this statement I also find that I can not entirely agree, for it is in just those places which are predisposed to decay through the acid action of the secretions and fermenting food particles on the obscure and inaccessible surfaces of the posterior teeth, and the labial surfaces of other teeth, that gutta-percha is of greatest value, because, as I supposed, it was impervious to the action of the secretions. To be sure, the material undergoes a change, and in time needs replenishing or renewing; but that it does offer good resistance to the oral fluids I feel sure.

In the making and setting of porcelain inlays the color question is perhaps as important and vexatious a problem as we have to solve. The search for a durable and colorless cement has brought out the cellulose preparations mentioned by Dr. Head, but they are, up to the present time, of limited value. For those cases where we most need a translucent cement—namely, on the approximal surfaces—they lack the necessary strength.

The integrity of the attachment of the inlay to the tooth has been proved to me on two occasions in a rather unpleasant way, by having a contour filling fractured by stress of mastication, leaving a part of the porcelain firmly attached in the cavity of the tooth. This proves conclusively that if the inlay is properly grooved and set, its attachment to the walls of the cavity is all that could be desired.

The question of the durability of an inlay is one of the first that would naturally come to the mind of an operator taking up the work, and should be well considered. The statement that "a porcelain inlay is as durable only as a cement filling" is almost too fallacious to be noticed; but even when it is granted that the cement seam between the porcelain and the enamel-wall is the weak point of an inlay, we all know that the fine line of cement offers such a small surface to the action of the oral fluids that its disintegration is necessarily very slow, and after a certain length of time seems to stop altogether when the fit of the inlay is close. And it has occurred to me that this may be due to the lodgment in these crevices of a sort of sediment, which would act as a protection to the cement beneath. When the seam is found open through disintegration of the cement, it is an easy matter to wash it out with alcohol and wipe in some fresh cement, which really gives this inlay a new lease of life.

Some of such inlays have been in the mouth for seven or eight years and show no signs of failing, and if asked how much longer they would last I should prophesy that they would remain as long again.

On the other hand, if it were known that the life of an inlay were limited to a period of three or four years, there would still be many cases where an operator would be justified in using them for their cosmetic value.

Dr. Head: I believe that amalgam does have an antiseptic power. I have known many cases where the amalgam bulged or shrunk away from the cavity to such an extent that it was easy to place the instrument between the margin and metal, but when that amalgam was trimmed level the filling still continued to preserve the teeth for years. I think this experience of mine is not an uncommon one.

Fillings made of gutta-percha base plate are practically impervious to the fluids of the mouth, but the ordinary gutta-percha filling material will generally, in the course of two or three years, become disintegrated on the surface. Some gutta-perchas, however, seem to have a tendency to become vulcanized in the mouth, and so last much better, taking on a flint-like structure.

I have been very much interested in the Doyle celluloid cement, and, while I can not give it an unqualified indorsement, I feel that it is well worthy of a trial. It is a liquid celluloid.

The cavity should be absolutely dry and well undercut. The filling should be dried and undercut. The cement should be wiped over the filling and at the same time put into the cavity, when it should be dried with hot air to a thick gelatinoid consistence. This being accomplished, the porcelain inlay should be put upon this gelatinoid cement and carried home with a hot instrument. In this way we drive off the excess of the solvent in the cement, which is probably one of the chief causes of its disintegration.

I can now recall four or five mouths in my practice in which the Doyle cement has been used with excellent results; where the translucency of the cement has enabled me to get results that would have been impossible with the ordinary oxid of zinc cement.

Dr. R. H. Hofheinz: In regard to the porcelain inlay, I feel that when it has left the hands of the enthusiast and the ignorant man it will certainly have a lasting place in dentistry. I have only reason to take issue with you on the question of the antiseptic property. There is no antiseptic property in any filling.

This is the day of gold fillings, either soft gold or cohesive gold, and if it is a complicated filling it is a great deal more difficult to get close adhesion than with either gutta-percha or cement. There is no antiseptic filling that is known to me. Why should porcelain as an inlay exert any antiseptic property whatever upon the surrounding enamel or dentin? I believe the distinction which Dr. Head has made is wrong. It is not the question of antiseptic property, but it is the question of relative skill which is needed in putting in a soft filling or a plastic filling and a cohesive gold filling.

In the experiments made by Professor Miller—the only ones of which I know—he speaks of copper amalgam having slight antiseptic power.

Dr. R. Ottolengui: Considerably over a year ago this Doyle cement was sent to me with the claim that it could be used as a filling material. I did not undertake to use it in that manner, but I did try it as a cement for porcelain fillings, and I must say that the first inlay which I set with it made me feel that the millennium was really at hand. The joint was absolutely invisible, because the inlay was one which, placed in the cavity without a cement, gave an invisible edge, and the edge was no more apparent when the Doyle cellulose was used than before.

But in my enthusiasm over this cement I set a number of inlays, with the result that in the course of from two to four weeks I had the pleasure of resetting some of them and of making others over again which had not only dropped from the cavities, but had been lost.

As an evidence that it was not the inaccurate fit of the inlay or incorrect shape of the inlay, those which were brought back to me were reset with phosphate cement, and are still in position. New ones made for the other cavities are also in position, set with cement.

In regard to the manner of using the cement, I used it exactly as Dr. Head describes, with the exception of the hot instrument. The appearance of a filling set with this cement is so good that I am willing to set one or two more and use the hot instrument to see, but I must confess that I am very skeptical as to its having any lasting utility.

Dr. Head seems to admit that it is not to be used in cavities which must endure any great strain, and Dr. Tracy makes the point that they might do very well in labial fillings, but not in approximal cavities.

Now, it is a curious thing how different the experiences of different operators are in this porcelain inlay matter. Nearly every one claims that the ideal place for porcelain inlays is in the labial surfaces, and very few, if any, seem to consider that the labial cavity is at all difficult. In the matter of retention, my own experience has been that ninety percent of my failures have been in labial cavities, whereas I do not remember to have lost a filling from an approximal surface which did not reach the masticating surface. One of the first of those I put in was of the old German glass filling material set with ordinary cement, and the patient eats on it and it stays there.

Dr. M. L. Rhein: After a varied use of porcelain for filling material and for other purposes during the past fifteen years, I must agree very strongly with the remarks of Dr. Capon, that we cán not at the present time look upon the porcelain inlay as a permanent affair.

While we all admire the beautiful porcelain work that Dr. Head has shown at different meetings, and recognize his ability to reach results in certain difficult cases, I feel that there is a danger of his taking a rather too extreme view of the value that we can hope to obtain at the present day from the use of porcelain, and at the same time I fear the effect of decrying the use of gold properly manipulated in the preservation of tooth structure.

With some little hesitation I have taken the floor to differ with Dr. Head on this account, but I feel personally very strongly, as Dr. Hofheinz did in the remarks he made on the subject, in regard to the differences that Dr. Head claims to find between the value of gold and amalgam. If there is any deterioration in the margins of such fillings—

an effect, by the way, which should never occur in a properly manipulated gold filling, but which may not be due to the defective filling after all—and even if such a thing should take place in regard to leakage, which would result in the deterioration of the dentin in a tooth with a gold filling, I thoroughly agree with the sentiment of Dr. Hofheinz that it is due entirely to poor manipulation of the gold in the cavity.

The amalgam, on account of the ease of manipulation, will adhere to the walls of the cavity. Too much attention has been given, in the last few years especially, to the ease with which gold can be placed in the teeth, without regard to its proper adaptation to the walls of the cavity. It is a point where the skill of the operator comes into play, and the blame can not always be attached to the material that is used.

The main object that I have in making these remarks is that whenever anything has come into vogue as rapidly as porcelain has been brought out, since the electric furnace has been so prominently and extensively used, there is danger of going to extremes in it. and attempting to accomplish something that we may afterward regret.

Wherever esthetic or cosmetic purposes are not a large desideratum, it still remains my humble opinion that we have no material that will preserve tooth structure as permanently and as thoroughly and that has so few objectionable qualities as a properly inserted gold filling.

The objection to this, as enumerated by Dr. Head, seems to be fraught with the great evil that is underlying operative dentistry at the present time, the desire to accomplish something that has been in vain sought for by the profession for a great many years—the insertion of a filling material without the expense of much labor to the operator. Up to the present time no one has solved the problem.

In the same light that I feel that a porcelain inlay can not save a tooth as permanently nor as well as a properly inserted gold filling, I feel that a gold inlay can not save teeth properly. I have seen a great many gold inlays inserted in teeth, and sometimes a most deplorable resulting condition, starting with the loss of the cement around them. I could see by the character of the work in many of them that they were well put in and well made. I knew that the man who made them was a good workman, but, as far as permanency goes, they bear the same relation to a properly inserted gold filling as a porcelain inlay would, being dependent entirely upon the imperfect and temporary character of the cement which holds the inlay.

Dr. Van Woert: I would like to speak generally rather than to either of the gentlemen in particular, and what I want to say most, and make most emphatic, is that those of you who have not taken up porcelain work had better be careful before you do.

I think I have spent as many hours in my laboratory as almost any gentleman here working in this same line, and have tried various materials. I have worked with the same people Dr. Head worked with, and the same materials, which I found very satisfactory, but I did not often choose a fusible inlay.

I would advise you, if you care to make the experiment, to examine the inlays of any man. If you can find any of mine, examine them with a magnifying glass, and you will find them very defective. In the first place, they have got to be firm in the matrix. That will change; therefore at some one point or other there is a bad place in the inlay. The cements that we have I do not believe are adequate or fit for the purposes to which they are put.

I spoke upon this subject in New Jersey last month, and one gentleman told of where he had used a certain kind of cement and it had lasted many years. I can show you cement fillings put in my wife's mouth which have been there for twenty-three years. I have several patients in whose teeth cement fillings were placed many years ago, and they are doing good service to-day; but I could not tell you the full reason for this It is evident that where a porcelain inlay is inserted into a tooth with space between it and the tooth, the space has got to be filled with some plastic material, and whatever that material is it is going to prove defective in some mouths.

Now, I am going to reiterate another statement I have made. I believe that porcelain inlays have come to stay. I believe that every dentist wants an outfit in his office. I believe that when he starts in he must be very careful where he places them, and with the understanding that there is a possibility of its proving a failure; and if it is a failure he must not be blamed. He is taking the same chance as the patient, and surely a great deal of hard work has been done to better the materials that we have at our hand. I do not believe that it is possible to fail in making an inlay that would be what you would call perfect with any furnace, but I think a good electric furnace is the best any man can use.

Porcelain inlays placed in the mouths of people as we have been doing in the past are going to be like lots of the copper amalgam fillings placed in years gone by, and like lots of the bridges in years gone by; and if we stick to this as we have begun we will be in the same position with regard to porcelain as an inlay. This porcelain work is now stepping in to relieve us of what was wrong in days gone by, and it is attracting the attention and the manipulative ability of some of the finest gold workers in the world. We have very few men who stick to gold filling in these days; they are all looking for something easy to do I am afraid to pass judgment, because I feel timorous myself; but I think a good deal of it is pure laziness.

I tell you, gentlemen, if you maintain the skill and standard of your profession, you will keep up your ability to manipulate gold, either in soft fillings, cohesive fillings or whatever it may be.

Dr. C. S. Butler: If I understood Dr. Capon aright, he advocates the use of porcelain work, among other things, for the reason that it develops a higher degree of skill and manipulative ability in the man who uses it; and it is upon just that point that I believe the enthusiasm over porcelain work will have a permanent benefit, even though porcelain inlays may sooner or later disappear. I believe that anything which has a tendency to develop the skill of the operator, anything that is hard to do, and requires study and perseverance to accomplish, is the very thing which is doing us good, though it may in itself be of a temporary character.

Whether porcelain inlays are to be permanent or whether they are to pass away, as was suggested by Dr. Van Woert, I do not know, but I do believe that the very fact that men in our profession are devoting to this work so much time and labor is developing a higher degree of manipulative ability in the profession, and consequently, instead of suffering in our manipulation of gold work and other materials, we will, as a profession, be developed to a higher degree of skill in all directions; and for this reason, if for no other.

Dr. Capon: There must be some body to the cavity that goes into the teeth, and not a mere shallow surface. I try to get as much impression as I can of the depth of the cavity. If I punch through my platinum matrix I can bridge over with porcelain, and it makes no difference to the filling when finished. Sometimes I even punch it through purposely, and force through a little bit of the body and make a little button, so to speak, to make a retention.

Now, with the thin veneer it can not get any retention. It will soon dislodge. I think depth of cavity is what is needed most. After I get the body of the cavity properly attended to and approximate the edges, then I make a biscuit with the higher grade body. I can approximate the contour in the biscuit. I replace it in the cavity, and then attend to the edges. The platinum is trimmed away until I have a thin margin of the metal to deal with instead of a large flap. The biscuit gives something to hold it into position while I burnish the edges.

Now, when that biscuited portion is taken out, it should be done so carefully; more carefully than the first time, and I think it should be taken out with a little lifting and coaxing. Do not go at it as if it were heavy material; go at it easily, a little bit at a time. Do not try to pull it out, or you will mar your edges or change them.

If the access to the cavity be easy and a large cavity, I will put in two or three porcelains while you are putting in a gold one, but I may take about half an hour to put in one of those that I call easy ones.

You should get sufficient depth to hold these inlays, even if the teeth are sensitive. There are means to obtain it, but without sufficient depth you can not claim any durability at all in porcelain inlays. Porcelain inlay is the part of the porcelain work to which I attach the least amount of importance, because my art lies in the crown. That is where I feel I do the best work and the most good to my patients.

I always use Close's body for my first biscuit. Then often a mixture of Close's and Whiteley's as an enamel.

I use as enamel, so to speak, either the Consolidated body or Dr. Whiteley's new body. They have given me as good satisfaction as any.

In regard to shading these, it becomes somewhat easy, and one who has experimented along this line will be able to get as many as ten or eleven different shades. Shading is not a difficult thing for me now. I do not always get them exact the first time, but I use the basal powders and change and mix them until I get what I want, just as an artist takes his palette and mixes the different colors.

The higher the grade the less shrinkage. The Close's body does not take as fine a finish as the others. You get less shrinkage, because the water will be taken away far more readily than it will with the finer ground bodies.

